# A novel closed-loop biotechnology for recovery of cobalt from a lithium-ion battery active cathode material

**DOI:** 10.1099/mic.0.001475

**Published:** 2024-07-17

**Authors:** Eva Pakostova, John Graves, Egle Latvyte, Giovanni Maddalena, Louise Horsfall

**Affiliations:** 1 Centre for Health and Life Sciences, Institute of Health and Wellbeing, Coventry University, Coventry, CV1 5FB, UK; 2 Centre for Manufacturing and Materials, Institute for Clean Growth and Future Mobility, Coventry University, Coventry, CV1 5FB, UK; 3 MIRARCO Mining Innovation, Sudbury, ON P3E 2C6, Canada; 4 Goodman School of Mines, Laurentian University, Sudbury, ON P3E 2C6, Canada; 5 School of Biological Sciences, University of Edinburgh, Edinburgh, EH9 3FF, UK; 6 Faraday Institution (ReLiB project), Quad One, Harwell Science and Innovation Campus, Didcot, UK

**Keywords:** bioleaching, cobalt electrowinning, closed-loop metal recycling, lithium-ion batteries, nanoparticles

## Abstract

In recent years, the demand for lithium-ion batteries (LIBs) has been increasing rapidly. Conventional recycling strategies (based on pyro- and hydrometallurgy) are damaging for the environment and more sustainable methods need to be developed. Bioleaching is a promising environmentally friendly approach that uses microorganisms to solubilize metals. However, a bioleaching-based technology has not yet been applied to recover valuable metals from waste LIBs on an industrial scale. A series of experiments was performed to improve metal recovery rates from an active cathode material (LiCoO_2_; LCO). (i) Direct bioleaching of ≤0.5 % LCO with two prokaryotic acidophilic consortia achieved >80 % Co and 90 % Li extraction. Significantly lower metal recovery rates were obtained at 30 °C than at 45 °C. (ii) In contrast, during direct bioleaching of 3 % LCO with consortia adapted to elevated LCO levels, the 30 °C consortium performed significantly better than the 45 °C consortium, solubilizing 73 and 93 % of the Co and Li, respectively, during one-step bioleaching, and 83 and 99 % of the Co and Li, respectively, during a two-step process. (iii) The adapted 30°C consortium was used for indirect leaching in a low-waste closed-loop system (with 10 % LCO). The process involved generation of sulfuric acid in an acid-generating bioreactor (AGB), 2–3 week leaching of LCO with the biogenic acid (pH 0.9), selective precipitation of Co as hydroxide, and recirculation of the metal-free liquor back into the AGB. In total, 58.2 % Co and 100 % Li were solubilized in seven phases, and >99.9 % of the dissolved Co was recovered after each phase as a high-purity Co hydroxide. Additionally, Co nanoparticles were generated from the obtained Co-rich leachates, using *Desulfovibrio alaskensis*, and Co electrowinning was optimized as an alternative recovery technique, yielding high recovery rates (91.1 and 73.6% on carbon felt and roughened steel, respectively) from bioleachates that contained significantly lower Co concentrations than industrial hydrometallurgical liquors. The closed-loop system was highly dominated by the mixotrophic archaeon *Ferroplasma* and sulfur-oxidizing bacteria *Acidithiobacillus caldus* and *Acidithiobacillus thiooxidans*. The developed system achieved high metal recovery rates and provided high-purity solid products suitable for a battery supply chain, while minimizing waste production and the inhibitory effects of elevated concentrations of dissolved metals on the leaching prokaryotes. The system is suitable for scale-up applications and has the potential to be adapted to different battery chemistries.

## Data Availability

Illumina sequence data used in this study are openly available in the European Nucleotide Archive (ENA) (accession no. PRJEB72310).

## Introduction

Lithium-ion batteries (LIBs) have higher energy, higher power densities, and a longer lifespan than other technologies. They have been broadly used in portable electronic devices (such as laptops and mobile phones) and electric/hybrid vehicles [1]. LiCoO_2_ (LCO) has been among the most extensively used active cathode materials in LIBs [2]. Both lithium (Li) and cobalt (Co) have been declared to be critical metals by the US government [3], the EU [4], and the UK government [5], due to their high economic importance and scarce supply; 75 % of Li is geographically concentrated in Argentina, Bolivia, and Chile (referred to as the ‘lithium triangle’) [6]. Further, primary Co production is highly susceptible to supply chain disruptions; 50 % of Co is mined in the Democratic Republic of Congo (DRC) and ~50 % of Co is refined in PR China [7]. The global supply is constantly threatened by political volatility, and many mining operations in the DRC are considered unethical due to violations of human rights, use of child labour in artisanal mines, and environmental negligence [8]. In response, transition metal oxides with a reduced Co content are used (LiNi_x_Co_y_Mn_z_O_2_), and a variety of Co-free materials have been developed, including LiFePO_4_ [9] and LiMn_2_O_4_ [10]. Especially under the pressure of the growing market for electric vehicles, the production of LIBs and subsequent accumulation of waste LIBs will continue. However, due to the competitive high energy density of LCO, which contributes to the smaller size of the devices [11], it still may take time before LCO can be completely displaced in LIBs. Moreover, the increasing usage of electronics has resulted in an already substantial discard stream of LCO.

Effective recycling strategies need to be developed to address the serious waste management challenges arising from the accumulation of end-of-life LIBs. Financial analyses show that LIB recycling is not economical unless significant Co and Ni contents can be recovered [12,13]. All current industrial processes that recycle waste LIBs use pyrometallurgy, hydrometallurgy, or a combination of the two approaches [14]. Bioleaching is a ‘green’ alternative to the conventional technologies, which are costly (due to the high energy consumption and use of expensive extraction solvents) and harmful to the environment (due to high emissions and use of harsh chemicals). Bioleaching is relatively simple, less demanding for energy, and has fewer negative environmental impacts. It has been used successfully for decades in base and precious metal recovery from sulfidic ores and gold concentrates [15,17]. Extremely acidophilic prokaryotes (with pH optima <3) accelerate oxidative dissolution of the sulfides via mechanisms that have been described previously [18, 19,20]. Rather than using individual species, it is advantageous to construct leaching consortia comprising of sulfur (S) and ferrous iron (Fe^2+^) oxidizers for biomining industrial processes [21]. Fe^2+^ oxidizers regenerate the primary chemical oxidant ferric iron (Fe^3+^) from Fe^2+^, while S oxidizers generate H_2_SO_4_ from reduced inorganic S compounds (such as sulfides). The latter process lowers the pH to values suitable for the growth of acidophiles, reduces Fe^3+^ precipitation, and supplies H^+^ for chemical attack on acid-soluble sulfides. Heterotrophs and mixotrophs, if present, prevent the accumulation of organic carbon (from cell exudates and lysates) that inhibit mineral oxidizers.

Despite the apparent advantages of bioleaching, the technology remains rather niche in the metal mining sector, mainly due to its slow kinetics. Bioleaching has been shown to have significant advantages in metal recovery from low-grade ores and historic mine tailings (that may still contain significant concentrations of valuable metals), neither of which is economical to process with a different technology. As mentioned above, end-of-life LIBs contain a variety of valuable metals, some of which (especially Co, Li, Mn, and Ni) can be present in high to very high concentrations [2], which presents a different set of challenges. Metals have been bioleached from spent LIBs and active cathode materials on a laboratory scale with various metal recovery rates (Table 1) using the fungus *Aspergillus niger* [22,25], as well as acidophilic chemolithotrophs in pure [25,28] or mixed cultures [29,34].

**Table 1.** Metal extraction rates during bioleaching of spent LIBs or active cathode materials using acidophilic chemolithotrophs or the fungus *Aspergillus niger*. Times in parentheses show the duration of biogenic lixiviant generation, while times marked with an asterisk show the duration of leaching phases during indirect leaching with biogenic lixiviants. Unmarked times show the total duration of bioleaching

Microorganism(s)	Battery material	Experiment type	Metal(s)	Extraction rate(s) (%)	Pulp density [% (v/w)]	Substrate(s)	Time (days)	Reference
**Acidophilic chemolithotrophs (pure cultures**)								
*Acidithiobacillus (At.) ferrooxidans*	Spent LIBs	Flask	Co; Li	65; 10	1	S^0^+ Fe^2+^	15; 4	[26]
Adapted *At. ferrooxidans*	Spent LIBs (mainly LCO)	Flask; Cu as catalyst	Co	99.9	1	Fe^2+^	6	[27]
Adapted *At. ferrooxidans*	Spent LIBs (mainly LCO)	Flask; Ag as catalyst	Co	98.4	1	Fe^2+^	7	[28]
*At. thiooxidans* strain 80 191	Spent LIBs	Flask; spent medium	Co; Li	22; 66	0.25	S^0^	40	[25]
*At. thiooxidans* strain 80 191	Spent LIBs	Flask	Co; Li	2.7; 22.8	0.25	S^0^	40	[25]
*At. ferrooxidans* strain DSMZ 1927	Spent LIBs	Flask; indirect; stepwise	Co; Li;Mn; Ni	90.4; 89.9; 91.8; 85.5	10	Fe^2+^	(7); 3×2 h*	[40]
**Acidophilic chemolithotrophs (mixed cultures**)								
Unspecified S- and Fe^2+^-oxidizing bacteria	Spent LIBs	Flask	Co; Li	90; 80	1	S^0^+ Fe^2+^	5	[34]
*At. ferrooxidans* and *At. thiooxidans*	LIBs	Flask	Co; Li	83; 85	1	S^0^+ Fe^2+^	35	[33]
*At. ferrooxidans* DSM 14882 and *At. thiooxidans* DSM 14887	Spent LIBs	Flask; indirect; stepwise	Co; Cu;Li; Mn;Ni	53.2; 74.4; 60; 81.8; 48.7	10	S^0^+ Fe^2+^	(N.A.); 4×1 h*	[56]
Adapted *At. ferrooxidans* and *At. thiooxidans*	Spent LIBs	Flask; concentrated spent medium	Co; Li; Ni	50.4; 99.2; 89.4	4	S^0^+ Fe^2+^	16	[30]
Consortium dominated by *Leptospirillum (L.) ferriphilum* and *Sulfobacillus (Sb.) thermosulfidooxidans*	LCO	Flask	Co; Li	100; 99.3	1.5	pyrite	3	[65]
*L. ferriphilum*-dominated consortium	Spent LIBs	Flask; two-step	Co; Li	99.36; 37.74	1	Fe^2+^	2 to 8	[31]
Adapted *At. caldus, L. ferriphilum, Sulfobacillus* spp*.* and *Ferroplasma* spp.	Spent LIBs (mixed crushed cathode and anode)	Flask	Co; Li; Ni	99.9; 84; 99.7	1	S^0^+ Fe^2+^	2	[29]
Consortium dominated by *L*. *ferriphilum* and *Sb*. *thermosulfidooxidans*	LCO	Flask; two-step; exogenous glutathione	Co; Li	96.3; 98.1	5	Pyrite	5	[32]
Adapted *Acidithiobacillus and Alicyclobacillus*-dominated consortium	Spent LIBs	Flask; two-step	Al; Co; Li; Mn; Ni	All 100	1	S^0^+ Fe^2+^	7	[76]
**Fungi**								
*Aspergillus(A.)niger* strain PTCC 5210	Spent LIBs	Flask; spent medium	Al; Co; Cu; Li; Mn; Ni	65; 45; 100; 95; 70; 38	1	Sucrose	16	[22]
*A. niger* strain PTCC 5210	Spent LIBs	Flask; one-step	Al; Co; Cu; Li; Mn; Ni	58; 0; 11; 100; 8; 0	1	Sucrose	16	[22]
*A. niger* strain PTCC 5210	Spent LIBs	Flask; two-step	Al; Co; Cu; Li; Mn; Ni	61; 1; 6; 100; 10; 0	1	Sucrose	16	[22]
*A. niger* strain PTCC 5210	Spent LIBs	Flask; spent medium	Al; Cu;Li; Mn	75; 100; 100; 77	2	Sucrose	8	[23]
*A. niger* strain PTCC 5210	Spent LIBs	Flask; spent medium	Co; Ni	64; 54	1	Sucrose	8	[23]
Adapted *A. niger*	Spent LIBs	Flask	Al; Co; Cu; Li; Mn; Ni	62; 38; 94; 100; 72; 45	1	Sucrose	30	[24]
Adapted *A. niger*	Spent LIBs	Flask; spent medium	Al; Co; Cu; Li; Mn; Ni	60; 15; 100; 63; 54; 20	1	Sucrose	(10)	[24]
*A. niger* strain MM1	Spent LIBs	Flask	Co; Li	67; 87	0.25	Sucrose	40	[25]
*A.niger* strain MM1	Spent LIBs	Flask; spent medium	Co; Li	82; 100	0.25	Sucrose	40	[25]

To recover solubilized metals, different recovery techniques can be applied. Solvent extraction (SX; using e.g. Cyanex 272, PC-88A, and D2EHPA) has been used to recover metals from LIB liquors, but organic solvents are expensive and would inhibit micro-organisms in biohydrometallurgical operations if metal-stripped liquors (raffinates) were recirculated. SX has been used in combination with electrowinning to recover Co from liquors originating from hydrometallurgical processing of polymetallic ores (e.g. [35]) and from bioleaching of a Co-rich pyrite concentrate [36], with SX securing Co concentrations of 40–50 g l^−1^ prior to electrowinning. On the downside, electrowinning is sensitive to perturbations (e.g. in solution composition), and in many instances, economic feasibility needs to be assessed. Precipitation is often used to recover metals from organic phase after SX [37,39], but selective precipitation can also be applied without prior use of solvents. Cobalt has been precipitated from chemical LIB leachates as oxalate (e.g. [40,42]) and hydroxide [43,45], while Li is often recovered as carbonate [41,43]. The efficiencies of metal recovery from solution in literature generally exceed~90 % for Co and 70 % for Li. Additionally, bioprecipitation can be used to recover metals from solution in the form of high-value nanoparticles, with the main advantages being low cost and bacteria serving as reusable catalysts in such systems. Sulfate-reducing bacteria (SRB; such as *Desulfovibrio* spp.) precipitate metals as sulfides (e.g. [46,48]) that can possess a variety of improved traits; Ni sulfide with unusual superparamagnetic and electrochemical properties [49,50], Zn sulfide quantum dots [51], and Pd with increased catalytic activity have been reported [52,53]. Recently, *Desulfovibrio alaskensis* has been reported to synthesise Co nanoparticles and subsequently recover the metal from chemical LIB leachates [54].

A vast majority of published reports that address the complete process route of Co extraction from LIBs and subsequent recovery of Co and/or Li products (from which LCO can be resynthetized, e.g. [55]), have applied chemical leaching. Boxall *et al*. [56] and Do *et al*. [40] applied indirect bioleaching using biogenic Fe^3+^ and H_2_SO_4_ to extract metals from a LIB waste. In both studies, low extraction rates were significantly improved by the engagement of a sequence of leach stages. To our knowledge, there are a very limited number of reports describing metal extraction from LIBs using microorganisms and subsequent metal recovery from the obtained bioleachates [25,40]. The primary objective of this work was to investigate bioleaching of metals from LCO by designed mesophilic and moderately thermophilic bacterial consortia. As the dissolution of active cathode materials happens predominantly via acid dissolution, the most suitable approach for the process is S-enhanced bioleaching (reviewed in [16]), during which elemental sulfur (S^0^) is externally supplied. S^0^ is cheap and produced in vast quantities as a waste product during e.g. natural gas processing. Bio-oxidation of S^0^ provides H_2_SO_4_ for acidic dissolution of LCO, maintains a low pH that is required for acidophiles to grow, and prevents metal precipitation. In this study, solubilization of Co and Li from 0.5 and 3 % (w/v) LCO using non-adapted and adapted bacterial consortia, respectively, was investigated. The adapted mesophilic bacterial consortium was used for indirect leaching in a closed-loop system that comprised the following steps: (i) generation of biogenic H_2_SO_4_ in an acid-generating bioreactor (AGB), (ii) indirect leaching of LCO with the biogenic acidic lixiviant, (iii) selective precipitation of Co as hydroxide, and (iv) recirculation of the Co-stripped liquor back into the AGB. Furthermore, electrowinning of Co from bioleachates was investigated and shown to be a viable alternative to selective precipitation.

## Methods

### Direct bioleaching of LCO using non-adapted cultures

A sterile medium (121 °C, 15 min) containing basal salts and trace elements [57] adjusted to pH 2.0 (using 1 M H_2_SO_4_) was aseptically distributed into four 200 ml conical flasks sealed with a foam stopper and Al foil. Each flask containing 90 ml medium was supplemented with 1 % S^0^ (sterilized at 105 °C, 30 min) and 20 mM Fe^2+^ (from a filter-sterilized 1 M FeSO_4_·7H_2_O stock solution, pH 1.8). Duplicate flasks were inoculated with 10 ml of prokaryotic consortia sourced from the Acidophile Culture Collection maintained at Bangor University (UK). The bacterial species in each consortium and their main physiological characteristics are shown in Table 2. LCO (powder SLC03007, Targray, Canada; D90=29.8 µm, purity 99.9 %, nonsterile) was added at 0.2 and 0.5 % (w/v) pulp density to duplicate cultures, which were then cultivated at 100 r.p.m. and 30 or 45 °C. Two sets of controls were used, uninoculated and LCO-free. All bioleaching systems were regularly sampled to monitor pH, *E*_H_, planktonic cell counts, and dissolved metal concentrations. Additions of sterile H_2_O prior to every sampling occasion compensated for evaporation. The direct LCO bioleaching experiment with non-adapted cultures continued for 28 days.

**Table 2.** Acidophilic chemolithotrophic bacteria used to leach metals from LCO

Bacterium	Temp. optimum(range) (°C)	pH optimum(range; minimum in bold)	S oxidation	Fe^2+^ oxidation(Fe^3+^ reduction)	Reference
**30 °C consortium**					
*Acidithiobacillus (At.) ferrooxidans* ^T^	28 –30(10–37)	2.0–3.0(**1.3**–5.5)	+	+ (+)	[84]
*At. ferrooxidans (‘ferruginosis’*) strain CF3	28–30(10–37)	~2.5(min. **1.3**)	+	+ (+)	[85]
*At. ferridurans* ^T^	29(10–37)	2.1(**1.4**–3.0)	+	+ (+)	[86]
*At. thiooxidans* ^T^	28–30(10–37)	2.0–3.0(**0.8**–4.0)	+	– (–)	[87]
*At. caldus* strain BRGM1	45(32–50)	2.0–2.5 (0.**8**–3.5)	+	– (–)	[84]
*Sulfobacillus (Sb.) thermosulfidooxidans* ^T^	50(20–58)	1.9–2.4(**0.8**–5.5)	+	+ (+)	[88]
*Leptospirillum (L.) ferrooxidans* ^T^	35(10–45)	1.6–2.0(min.~**1.0**)	−	+ (+)	[89]
*Alicyclobacillus* sp.(‘*disulfidooxidans’*)	35(4–40)	1.5–2.5(**0.5**–6.0)	+	+ (+)	[90]
**45 °C consortium**					
*At. caldus* strain BRGM1	45(32–50)	2.0–2.5 (0.**8**–3.5)	+	– (–)	[84]
*Sb. thermosulfidooxidans* ^T^	50(20–58)	1.9–2.4(**0.8**–5.5)	+	+ (+)	[88]
*Sb. acidophilus* strain BOR4	45–50(na)	2.0–2.2(**~1.0** to 3.0)	+	+ (+)	[75]
*Acidithiomicrobium* strain P2	45–50(max. 58)	~2.0(min.~**1.0**)	+	+ (+)	na

### Direct one-step and two-step bioleaching of LCO using adapted consortia

LCO-resistant consortia were obtained via serial sub-culturing in a medium supplemented with S^0^ and Fe^2+^ as electron donors (as described above) and increasing concentrations of LCO [0.5, 1, 1.5, 2, and 3 % (w/v)]. All flasks were cultivated at 30 or 45 °C and 100 r.p.m. For adaptations to ≤1.5 % LCO, a one-step process was used, with the consortia being sub-cultured to a fresh medium with an increased LCO concentration when the pH dropped to ~1.0 and planktonic cell counts reached ~7×10^8^ cells ml^−1^. Two-step adaptation was applied to pulp densities of 2 and 3 % (w/v) LCO. (i) The cultures were pre-grown for 14 days in the absence of LCO. During this time, pH in mesophilic cultures dropped to ~1.1 and planktonic cell counts reached 2×10^8^ cells ml^−1^, while pH in the moderately thermophilic cultures reached 1.0 and cell counts increased to 4×10^8^ cells ml^−1^. (ii) Then LCO was added to each culture, and bioleaching was conducted as described above. For comparison, one-step bioleaching with consortia adapted to 3 % (w/v) LCO was conducted. The bioleaching processes with adapted cultures were monitored for 57 days (with active leaching by the 30 °C consortium for 16–20 days).

### Closed-looped indirect leaching of LCO with biogenic sulfuric acid

**Fig. 1.**
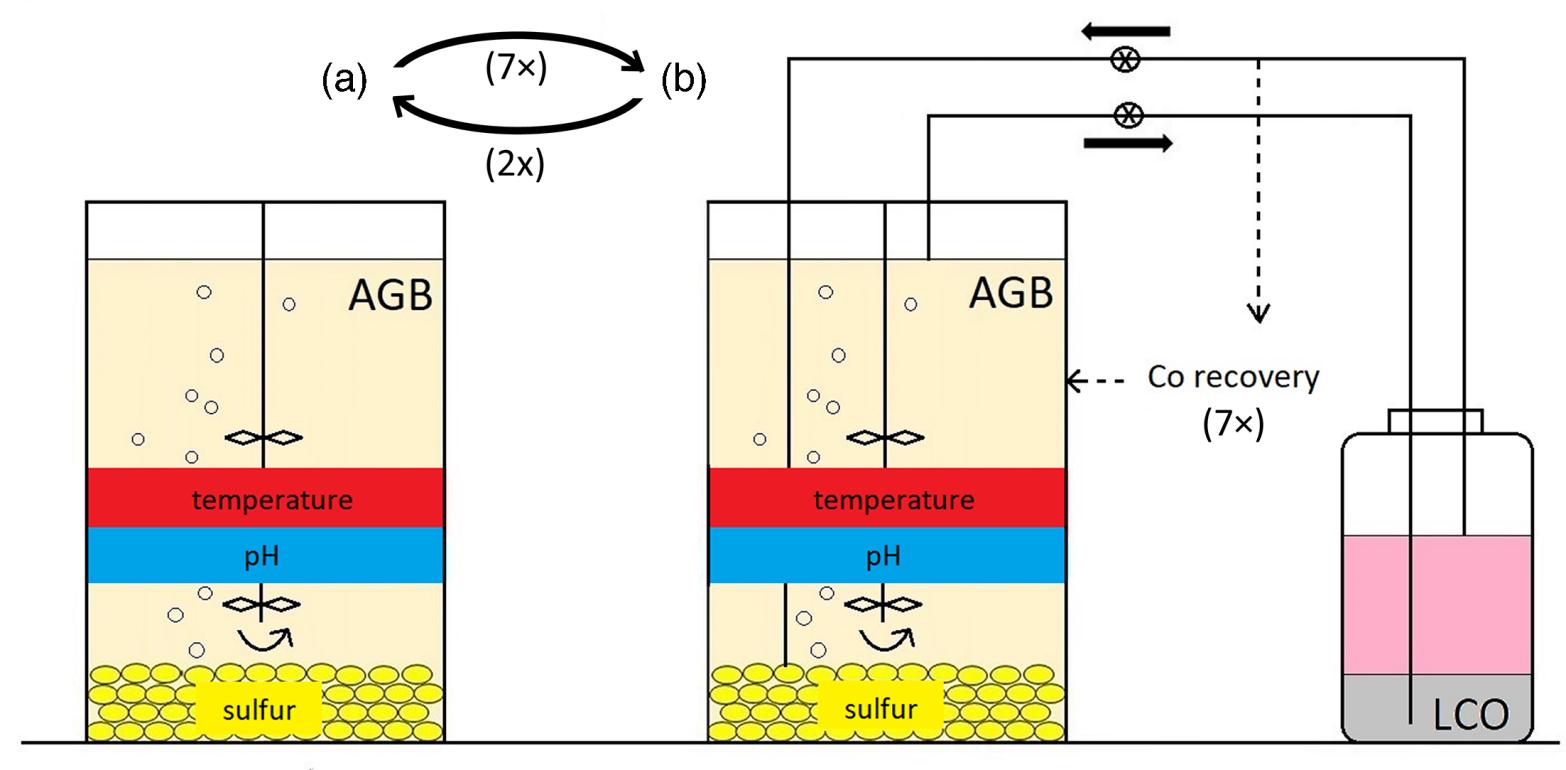
A schematic representation of the set-up used for closed-loop indirect leaching of LCO using lixiviants generated in an acid generating bioreactor (AGB). (**a**) The AGB was kept in a batch mode until the target pH of 0.9 was reached (sub-phases A); (**b**) LCO was continuously leached in sub-phase B, which was terminated by recovering of the dissolved Co. A new sub-phase A was initiated by recycling of an amended Co-free liquor (pH adjusted to 3.0 or 1.6 with H_2_SO_4_; supplemented with salts for microbial growth) or supplying a fresh basal salts medium into the AGB. The leaching phases (each comprising sub-phases A and B; their durations are shown in Table 3) were repeated until Co leaching ceased.

The closed-loop indirect leaching system (shown schematically in Fig. 1) involved seven repeating phases, each of which comprised sub-phases A and B (described below). The laboratory set-up of the closed-loop indirect leaching system is shown in Fig. S1, available in the online version of this article.

**Table 3.** Duration of individual sub-phases during indirect leaching of LCO. The duration of each sub-phase A was defined by the time needed for the pH inside the AGB (operated in a batch mode) to reach 0.9, after which indirect leaching of LCO (sub-phase B) commenced. Each sub-phase B ended with the recovery of dissolved Co from leachates, and a new phase commenced with the return of the raffinate (phases marked with asterisks) or the supply of a fresh basal salts medium (unmarked phases) into the AGB

	Phase duration (days)						
	**Phase 1**	**Phase 2**	Phase 3*	**Phase 4**	Phase 5*	**Phase 6**	**Phase 7**
**Sub-phase A**	12	14	16	14	14	12	8
**Sub-phase B**	13	13	21	18	12	20	16

#### Set up and operation of sulfuric acid-generating bioreactor

An AGB was set up to generate biogenic H_2_SO_4_ from S^0^ using the mesophilic acidophilic consortium adapted to 3 % LCO. The system (Fermac 360, Electrolab, UK) comprised a 2 l vessel coupled to pH and temperature control modules. The reactor vessel was heat-sterilized (121 °C, 15 min). After cooling down, 200 g of sterile (105 °C, 30 min) granular S^0^ (>99.9 % purity; J.R. Birchley, UK) was added to the vessel, followed by 920 ml of sterile basal salts medium (pH 2.5) supplemented with trace elements [57] and 80 ml of inoculum. The vessel was connected to a sterile air supply (~1 l min^−1^) and maintained at 32 °C [except for phases 1 and 2 (described below) during which the temperature in the vessel was maintained at 35 and 30 °C, respectively]. The production of biogenic H_2_SO_4_ in AGB was monitored by regular pH readings and planktonic cell counts.

#### Sub-phase A: microbial generation of lixiviant

The indirect leaching of LCO was performed in seven phases (each comprising sub-phases A and B; Table 3), to limit the inhibitory effects of elevated Co concentrations on the leaching bacteria. Each leaching phase was initiated by a sub-phase A in which the AGB was operated in a batch mode (under the conditions described above) until the pH reached 0.9 (Fig. 1a).

### Sub-phase B: circular indirect leaching of LCO with biogenic lixiviant

When the pH in AGB reached 0.9, a non-aerated column (250 ml Duran bottle) containing 100 g of LCO (97 % purity; Alfa Aesar, USA) was connected to the AGB vessel. To initiate LCO indirect leaching, 250 ml of the acidic lixiviant generated in AGB was transferred to the column that was maintained at room temperature and 100 r.p.m. LCO was continuously leached with biogenic acidic lixiviant and leachate was recycled (Fig. S1) using an integrated peristaltic pump set to maintain the pH in thr AGB at 0.9. Volumes in the AGB and column were kept constant at 750 and 250 ml, respectively, and additions of sterile H_2_O to the column compensated for evaporation losses. The system was partially drained (approximately 700 ml of leachate from AGB and 200 ml from the column) every 2 to 3 weeks of indirect leaching. The dissolved Co was recovered by precipitation or electrowinning (described below). To minimize waste, the Co-free liquors were pooled together and supplemented with basal salts, trace elements (both in [57]) and 1 mM Fe^2+^. The pH of the amended raffinate was adjusted to 3.0 (first raffinate) or 1.6 (subsequent raffinates; using H_2_SO_4_) before it was returned into AGB, completing a sub-phase B and initiating another sub-phase A of closed-loop leaching. To avoid inhibitions of the prokaryotic leaching consortium by high Na concentrations (resulting from Co recovery using NaOH; see below), raffinates were recycled just once (i.e. after phases 3 and 5). After phases 1, 2, 4, and 6, a fresh basal salts medium was supplied into the AGB (as opposed to amended raffinate recycling).

### Chemical analyses of leachates

During all leaching experiments, pH and redox potential (relative to a standard hydrogen electrode, *E*_H_) were regularly monitored in leachate samples using a combination Ag/AgCl pH and a redox electrode (both Thermo Scientific Orion; Thermo Fisher Scientific, USA), respectively, connected to a benchtop pH/conductivity meter (Orion Versa Star Pro; Thermo Fisher Scientific, USA). Concentrations of Co and Li were measured in filtered (0.22 µm) leachates by inductively coupled plasma atomic emission spectroscopy (ICP-OES; Optima 8300; PerkinElmer, USA). Concentrations of Fe^2+^ and total Fe (after reducing soluble Fe^3+^ to Fe^2+^ with ascorbic acid) were determined using the ferrozine colorimetric assay [58]. Concentrations of Fe^3+^ were determined from differences between those of total Fe and Fe^2+^.

### Recovery of dissolved Co

#### Selective precipitation

After the first five leaching phases (all phases summarized in Table 3), Co was selectively precipitated (Fig. S2) from the leachates withdrawn from the AGB (700 ml) and column (200 ml). The column leachate was filtered (6 µm filter paper; Whatman, UK) to remove residual LCO. The pH of each leachate was adjusted to ~3.0 using NaOH (pellets and 1 M solution). The leachate was filtered (0.22 µm CA filter units; Corning, Inc., USA) to remove any Fe present, after which the pH of the filtrate was adjusted to pH 9–10 using NaOH. The formed Co precipitate was separated (0.22 µm CA; Corning, Inc., USA) from the filtrate, dried overnight at 75 °C, and weighed. The precipitate purity was analysed using ICP (after redissolving in dilute H_2_SO_4_, pH 1.3) and XRF (X-ray fluorescence; Rigaku, Nex DE, Japan). Additionally, the precipitation products obtained after phase 2 were washed twice with hot ultrapure water (pH ~10, adjusted with NaOH) before drying, and their purity was analysed. The Co-stripped liquors were sampled for a later ICP analysis that determined residual concentration of dissolved metals.

#### Electrowinning

Dissolved Co in the leachates collected after phases 6 and 7 was recovered by electrowinning (Fig. S3). First, the liquors collected from the AGB and leaching column were mixed (together with residual leachates from direct bioleaching), then the pH was adjusted to ~4.0 using NaOH, and the leachates were filtered (0.22 µm CA; Corning, Inc., USA). The volume of each leachate was adjusted to 900 ml before electrowinning: (i) carbon felt (99 %; 6.35 mm thick; Thermo Scientific Alfa Aesar, UK) was used for Co recovery from 6B bioleachate, and (ii) stainless steel 316 (1.6 mm thick; RS Components, UK) with a roughened surface was used for electrowinning from 7B bioleachate. For each Co deposition (Fig. S3A), a cathode was placed in a stirred tank between two mixed metal oxide anodes (De Nora, Italy), at a current density of 30 mA cm^−2^ and a temperature of 70 °C, with regular pH adjustments (every 12 min) to maintain pH between 2.6 and 4.2. To avoid deposition of metal hydroxides on the electrodes, the cathode and anodes were removed until the pH adjustment was finished. To monitor Co deposition, samples were withdrawn every 12 min, filtered and stored for future ICP analysis. The deposits were washed in hot ultrapure water, dried overnight at 75 °C, and examined by scanning electron microscopy (SEM; Carl Zeiss 1530 VP FEGSEM, Zeiss, Germany). The accelerating voltage used for secondary electron imaging was 5 kV. The surface morphology was examined on the final product after electrodeposition. Energy-dispersive X-ray spectroscopy (EDX) of the electroplated deposit was carried out using an Oxford Instruments X-Max 80 mm^2^ EDX detector with an accelerating voltage of 20 kV. The carbon felt sample was sputtered with an Au/Pd coating to improve conductivity for imaging.

#### Bioprecipitation of Co nanoparticles

Cobalt nanoparticles were generated from two Co-rich leachates (collected during direct LCO bioleaching and phase 2 of indirect LCO leaching) using SRB. *D. alaskensis* G20 was grown in Postgate C medium [59] at 30 °C in an anaerobic chamber (Whitley A95 Workstation, Don Whitley, UK) in an N_2_ atmosphere enriched with 10 % CO_2_ and 10 % H_2_. *D. alaskensis* cells were grown to an optical density (OD_600_) of 0.8–1.0, harvested by centrifugation (4000 r.p.m., 15 min), washed twice and resuspended [both in 10 mM 3-(N-morpholino)propanesulfonic acid buffer (MOPS), pH 7.5] to a final OD_600_ of 0.8–1.0. To generate Co nanoparticles, 1.8 ml of the suspension of washed cells was incubated under the culture conditions described above with 0.2 ml of amended bioleachates (obtained by Fe removal at pH 4.1, using NaOH, followed by filtration and finally dilution in double-distilled water; composition of amended bioleachates is shown in Results). After 20 h of incubation, samples for ICP-OES analysis were collected. Samples for determination of a total Co fraction were digested in HNO_3_ (20 %, 80 °C, 5 h), while samples for the quantification of dissolved Co were prepared by the removal of solids by centrifugation (16 000 r.p.m., 4 °C, 2 h). To determine the amount of Co recovered, the dissolved Co fraction was subtracted from the total Co fraction.

The nanoparticles formed were observed in assay samples drop-cast onto a C-coated Cu grid using transmission electron microscopy (TEM; JEM-1400 Plus EM, JOEL, USA), together with scanning transmission electron microscopy (STEM) and EDX (both Crossbeam 550, Zeiss, Germany).

### Microbiological and biomolecular analyses

Planktonic cells in the leachates and AGB lixiviant were regularly enumerated using a Thoma counting chamber and a Motic Panthera microscope (Motic Europe, Spain), at 400× magnification. Culturable mesophilic acidophiles were investigated throughout the 30 °C direct bioleaching experiments and during the indirect leaching (at the end of each sub-phase) by plating onto selective solid media [60]. All plates were cultivated at 30 °C for 14 days and isolates were identified by PCR using 27F/1492R primers, Sanger sequencing and blast search.

At the end of the bioleaching processes, the biomass was harvested from samples by filtration (0.22 µm). Genomic DNA was extracted using the DNeasy PowerSoil Pro kit (Qiagen, Inc., Germany), following the manufacturer’s protocol. DNA concentration and quality were determined using a NanoDrop 1 spectrophotometer (Thermo Fisher Scientific, USA). Extracted DNA was stored at −20 °C prior to submission for 16S rRNA gene amplicon sequencing. The PCR amplification of the V4 region using the 515F/806R primer pair [61], library preparation, Illumina MiSeq paired-end sequencing, and data processing were performed by the Centre for Environmental Biotechnology at Bangor University (UK) and have been described previously [62]. In short, the dual-indexing sequencing methodology was combined with the use of a heterogeneity spacer on the primer design to improve the quality of the reads. Sequences were pre-processed to joint pair-end reads, trimmed and cleaned, and barcodes were removed. The pre-processed reads were further processed using the pipeline developed by Fadrosh *et al*. [63] and the DADA2 plugin for Qiime2 (v2021.2) for denoising and ASV picking. Amplicon sequence variant (ASV) tables were generated and taxonomy was assigned using the Silva database (v. 138).

## Results

### Direct bioleaching of LCO using non-adapted cultures

Maximum metal recovery rates were obtained after 18 days of bioleaching of metals from 0.2 % LCO and after 22 to 25 days from 0.5 % LCO using non-adapted bacterial consortia (Table 4). Over 80 and 90 % of Co and Li were leached, respectively, from low amounts of LCO (0.2 and 0.5 % LCO pulp density) using the 45 °C consortium. In comparison, significantly lower metal recovery rates were observed during bioleaching with the non-adapted 30°C consortium, and these were negatively affected by the increasing pulp density (Table 4). Final pH values were not affected by the consortium type or pulp density, dropping from an initial pH 2.5 to ~0.8 in all cultures. The pH values in the LCO bioleaching assays reflected primarily two processes, the generation of H_2_SO_4_ via sulfur biooxidation and the consumption of H^+^ on metal dissolution from LCO. The increase of planktonic cell counts in all cultures from 10^7^ to 7×10^8^ cells ml^−1^ indicated microbial growth, and *E*_H_ values increased from +610 to +850 mV after 7 days of cultivation (reflecting primarily iron oxidation).

**Table 4.** Metal leaching from low LCO pulp densities using non-adapted prokaryotic consortia at 30 and 45 °C. Values are means of metal recovery rates obtained in duplicate cultures. Bacterial species and their metabolic traits included in each consortium are summarized in Table 2

	LCO pulp density (%)	Co leached (%)	Li leached (%)
**30 °C consortium**	0.2	77.8	83.3
	0.5	40.4	54.6
**45 °C consortium**	0.2	89.9	91.2
	0.5	82.7	95.4

### Direct bioleaching of LCO using adapted consortia

**Fig. 2.**
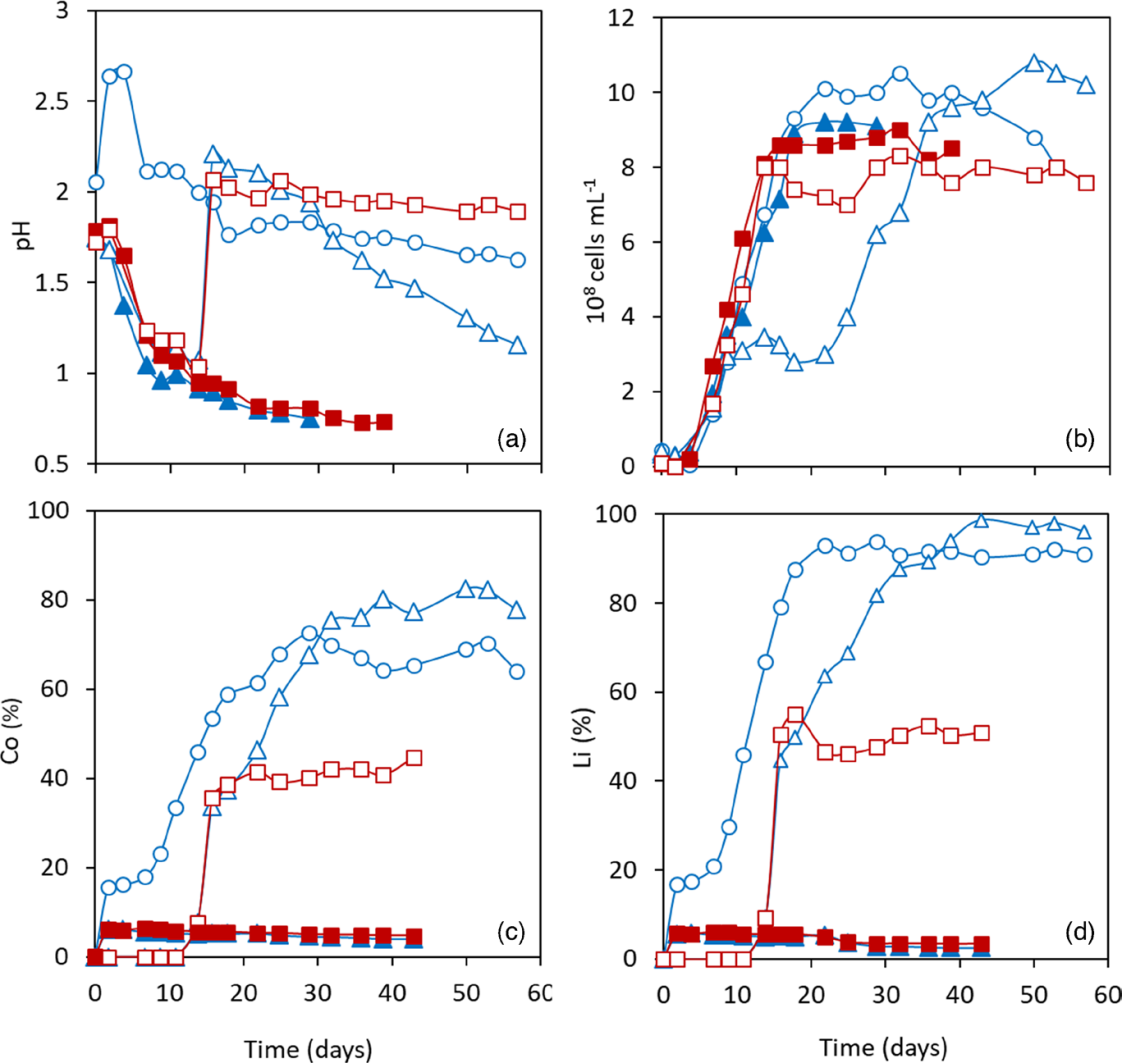
Changes in (a) pH, (b) planktonic cell counts, and concentrations of dissolved (c) Co and (d) Li during direct bioleaching of 3% LCO using adapted acidophilic prokaryotic consortia. 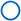 one-step bioleaching using 30 °C consortium 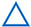 two-step bioleaching using 30 °C consortium; 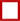 two step bioleaching using 45 °C consortium. Closed symbols show LCO-free (a,b) or uninoculated controls (c,d) at 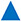 30 °C and 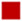 45 °C. Means of duplicate cultures are shown.

The 30 and 45 °C consortia were stepwise adapted to increasing LCO levels, with each subculture taking ~30 days for ≤1 % LCO and up to 60 days for LCO ≥2 %. The extended adaptation times with elevated pulp densities were ascribed to the toxicity of increased dissolved metals as well as the alkaline nature of LCO, resulting in an increase in pH (~2.7 for 3 % LCO; Fig. 2a) after LCO addition.

Unlike during direct bioleaching of low LCO pulp densities (0.2 and 0.5 %) with non-adapted cultures, the adapted 30°C consortium performed significantly better than the adapted 45 °C consortium, both during one- and two-step bioleaching of 3 % LCO (Fig. 2). During one-step bioleaching, only ~17 % of the Co and ~20 % of the Li were extracted at 45 °C and no changes in bacterial growth (~10^7^ cells ml^−1^), pH (3.6), and *E*_H_ (+560 mV; with Fe removed from solution as Fe^3+^ precipitates) were detected (data not shown), while the adapted 30 °C consortium solubilized 72.5 % of the Co (Fig. 2c) and 92.9 % of the Li (Fig. 2d). Additionally, bacterial growth equal to that of an LCO-free control (up to 10^9^ cells ml^−1^; Fig. 2b) and gradual decrease in pH (from 2.67 to 1.63; Fig. 2a) were observed in the duplicate mesophilic cultures. *E*_H_ in the one-step 30 °C-leaching system ranged between +725 and +790 mV (data not shown; with all iron present as Fe^3+^). Dissolution of Li and Co of about 6 % were detected in non-inoculated controls at both temperatures (Fig. 2c, d), indicating chemical dissolution of the metals was minimal. The final pH in LCO-free cultures reached values as low as 0.73 (Fig. 2a), and planktonic cell counts were 9×10^8^ cells ml^−1^ (Fig. 2b).

Improved metal solubilization was obtained (with both adapted consortia) during two-step bioleaching, compared to the one-step bioleaching systems. The maximum Co recovery rates were 44.8 and 82.5 % with the 45 and 30 °C consortia (Fig. 2c), respectively. Lithium extraction was even greater, reaching 55.1 and 98.7 % in the 45 and 30 °C-systems (Fig. 2d), respectively. The addition of LCO to pre-grown cultures (pH ~1.0, Fig. 2a; cell densities of 8×10^8^ and 3.5×10^8^ cells ml^−1^ for the 45 and 30 °C consortia, respectively, Fig. 2b) resulted in an increase in pH to ≥2.1 (Fig. 2a) and a slight drop in planktonic cell counts (Fig. 2b). The planktonic cell counts in the 45 °C consortium gradually recovered to the initial value, and those in the 30 °C consortium resumed growth after about a week after the LCO addition (Fig. 2b). The final pH in the two-step bioleaching systems was 1.90 at 45 °C and 1.16 at 30 °C (Fig. 2a). In all two-step bioleaching systems, *E*_H_ increased from values around +600 mV (ranging from+590 to +630 mV) to values between +740 and +800 mV (data not shown).

Plating onto selective solid media identified the S-oxidizing bacteria *At. thiooxidans* and *At. caldus* in samples collected during direct leaching at 30 °C (Table 5), while no Fe^2+^-oxidizing species were detected. Illumina amplicon sequencing of 16S rRNA genes indicated a dominance of *Ferroplasma* (93.8 % of total reads), followed by *Acidithiobacillus* (3.3 %), *Cuniculiplasma* (2.0 %), and *Acidibacillus* spp. (0.8 %). The 45 °C consortium was highly dominated by the genus *Sulfobacillus* (99.3 %), with a low proportion of *Leptospirillum* (0.4 %).

**Table 5.** Culturable organisms identified by plating onto solid media during direct bioleaching of 3 % LCO using an adapted 30 °C consortium. Shaded areas mark the presence of LCO in the leaching assay

Time (days)	0 % LCO	One-step bioleaching	Two-step bioleaching
1	T	T	T
7	T, C	T	T, C
14	T	na	na
32	–	T, C	C
50	–	T	C
60	–	T	T

T, *At. thiooxidans*; C, *At. caldus*; na, not available.

### Closed-loop indirect leaching of LCO

#### Process kinetics and metal leaching

The inhibition of leaching bacteria during direct bioleaching, even at low pulp densities, is a major limitation in bioleaching applications recovering valuable metals from spent batteries (or other electric and electronic wastes). Indirect leaching minimizes these inhibitions and enables processing of elevated loads of metal-bearing source materials. The closed-loop indirect leaching system designed to extract metals from 100 g LCO [an equivalent of 10 % (w/v)] was operated for 203 days, out of which the sum of duration of sub-phase A (acidic lixiviant generation) was 90 days and sub-phase B (LCO leaching and raffinate recirculation) was 113 days.

**Fig. 3.**
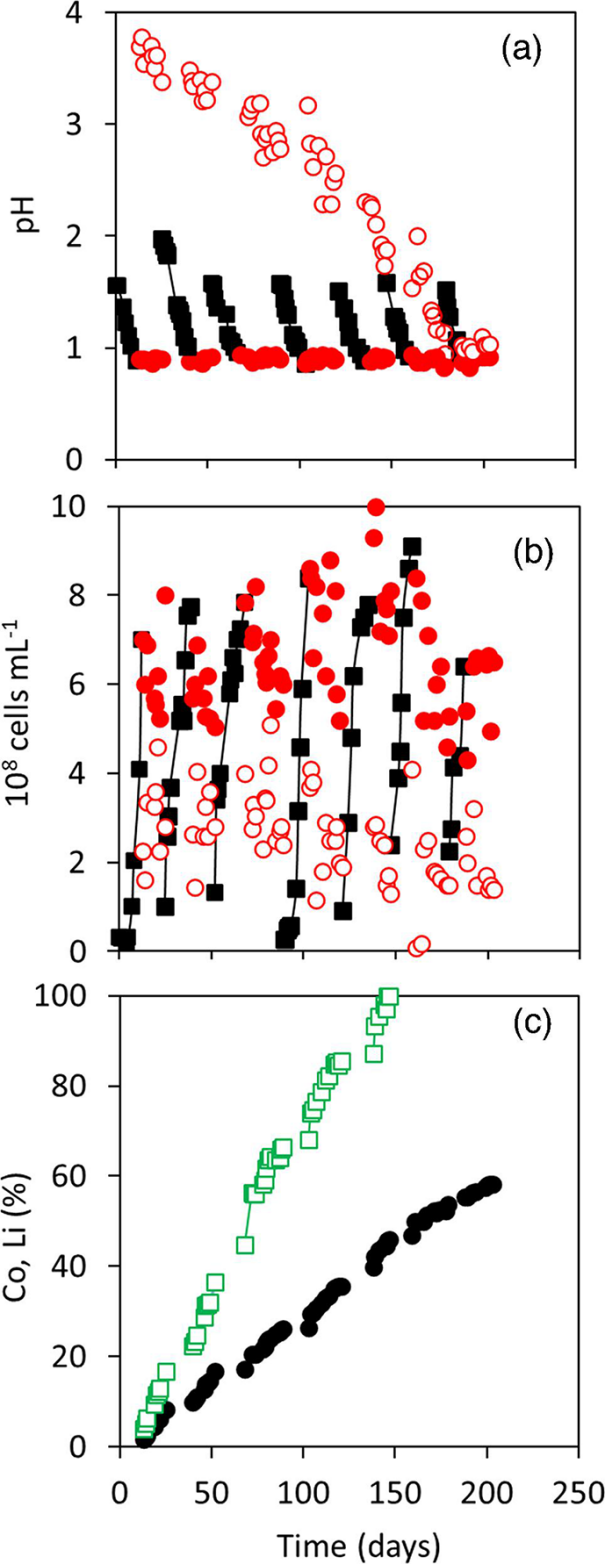
Changes in (**a**) pH and (**b**) planktonic cell counts during closed-loop indirect bioleaching of LCO using adapted mesophilic acidophiles. 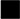 sub-phase A in AGB; 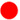 sub-phase B in AGB; 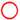 sub-phase B in leaching column. (**c**) Accumulated percentages of 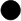 Co and 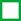 Li solubilized during indirect leaching (in the whole system).

The below parameters were monitored to assess process kinetics and leaching efficiency. Importantly, the development of these process parameters throughout the experiment provided insights into the bioleaching mechanism, and the limitations and opportunities of the application (see also Discussion). Fig. 3a summarizes pH measurements in AGB (during sub-phases A and B) and in the leaching column (sub-phase B). The dissolution of Li from LCO resulted in elevated pH values in the leaching column, with a maximum value of ~3.8 at the beginning of the first leaching sub-phase (1B), while the pH in AGB was maintained constant (at 0.9) by automated additions of the less acidic leachate. With a gradual depletion of Li, the pH in the leaching column gradually decreased to values as low as ~1.0 (in sub-phase 7B), at which point the leaching process stopped. Planktonic cell counts were monitored throughout the time course of the experiment (Fig. 3b), showing significantly higher numbers (*t*-test; *P*<0.05) in AGB (mean of 6.6×10^8^ cells ml^−1^) than in the leaching column (2.6×10^8^ cells ml^−1^).

Cumulative percentages of Co and Li leached from LCO are shown in Fig. 3c. All Li (>99.9 %) was extracted in the first five phases (with a rate of 90.4±10.6 mg day^−1^; mean±sd) and 58.2 % Co was solubilized within the time course of the experiment (in seven phases). Due to Li depletion in the last two leaching phases, the solubilisation of Co also slowed down. For comparison, between 8.3–10.3 % of total Co was leached in the first five phases (depending on the phase duration), while only 7.8 and 4.4 % Co was solubilized in phases 6 and 7, respectively. The mean rate of Co dissolution was 376.4±94.0 mg day^−1^ (mean±sd) in the first five phases, and 234.4 and 165.0 mg day^−1^ in the following two phases.

#### Co recovery from leachates

Following bioleaching, the recovery of metals from solution is required in solid forms suitable for the metal supply chains. It is desirable to achieve complete metal recovery in the form of valuable metal products of high purity. Selective precipitation using NaOH (Fig. S2) proved to be an efficient Co recovery technique, removing ≥99.8 % Co as hydroxide in all phases, with varying purity (Table 6a). The Co content in dried Co products declined from ≥96.7 % in phase 1B to as low as 83.2 % in phase 2B (and even lower in the more progressed phases), due to elevated Na content in the recovered solids. However, washing the filtered precipitates with hot water significantly improved their purity, which increased to ≥99.1 % after two rounds of washing of the solid products from phase 2B (Table 6a). Additionally, the washing procedure removed residual S^0^ when minor carryover from AGB to the leaching column occurred.

**Table 6.** Summary of Co recovery rates by (a) precipitation (using NaOH) from leachates collected from sub-phases 1B to 5B and (b) electrowinning (pH maintained at~4, 70 °C, 1 h) from leachates collected from sub-phases 6B and 7B. The purity of precipitation products is also reported, with values in parentheses showing Co hydroxide purity after the first and second round of washing, respectively

(A) Co precipitation				
**Phase**	**Leachate origin**	**Co in leachate (mg l^−1^**)	**Co recovery rate (%**)	**Co hydroxide purity (%**)
1B	AGB	4210	99.9	96.7
	Column	5910	99.8	98.4
2B	AGB	4258	100.0	89.6(97.2; 99.1)
	Column	5867	100.0	83.2(96.9; 99.5)
3B*	AGB	4964	100.0	92.2
	Column	7789	100.0	91.4
4B	AGB	5399	100.0	77.0
	Column	6139	100.0	81.2
5B*	AGB	5219	100.0	92.2
	Column	8823	100.0	91.4

(B) Co electrowinning:				
Phase	Cathode material	Co in leachate (mg l^−1^)	Co recovery rate (%)	
6B	Carbon felt	4020	91.1	
7B	Roughened steel	3294	73.6	

*, sub-phases that commenced with recirculation of Co-stripped liquors.

The conditions for Co electrodeposition were optimized for two different cathode materials. When a carbon felt cathode was used to recover Co from a bioleachate of pH 4.0 (adjusted before electrowinning using NaOH, and without further pH adjustments), only 54.1 % Co was recovered in the first hour of the deposition (with a current efficiency of 31.0 %). Extending the deposition time resulted in a higher overall Co recovery (76 % in 3 h) but lowered the current efficiency (to 14.6 %).

Significantly higher Co recovery rates were achieved with frequent pH adjustments that were performed every 12 min (Table 6b); 91.1 % of the dissolved Co was deposited on a carbon felt cathode (Fig. S3B; current efficiency 64.7 %) and 73.6 % on a stainless steel cathode with a roughened surface (Fig. S3C; current efficiency 66.8 %). Current efficiency fluctuated with decreasing Co concentration in the leachate (with the maximum obtained being 83.5 %), indicating that improved results could be achieved by strict control of the electrowinning process. Scanning electron micrographs and EDX spectra of the recovered Co deposits are shown in Fig. S4.

#### Evolution of prokaryotic consortium during closed-loop leaching

**Fig. 4.**
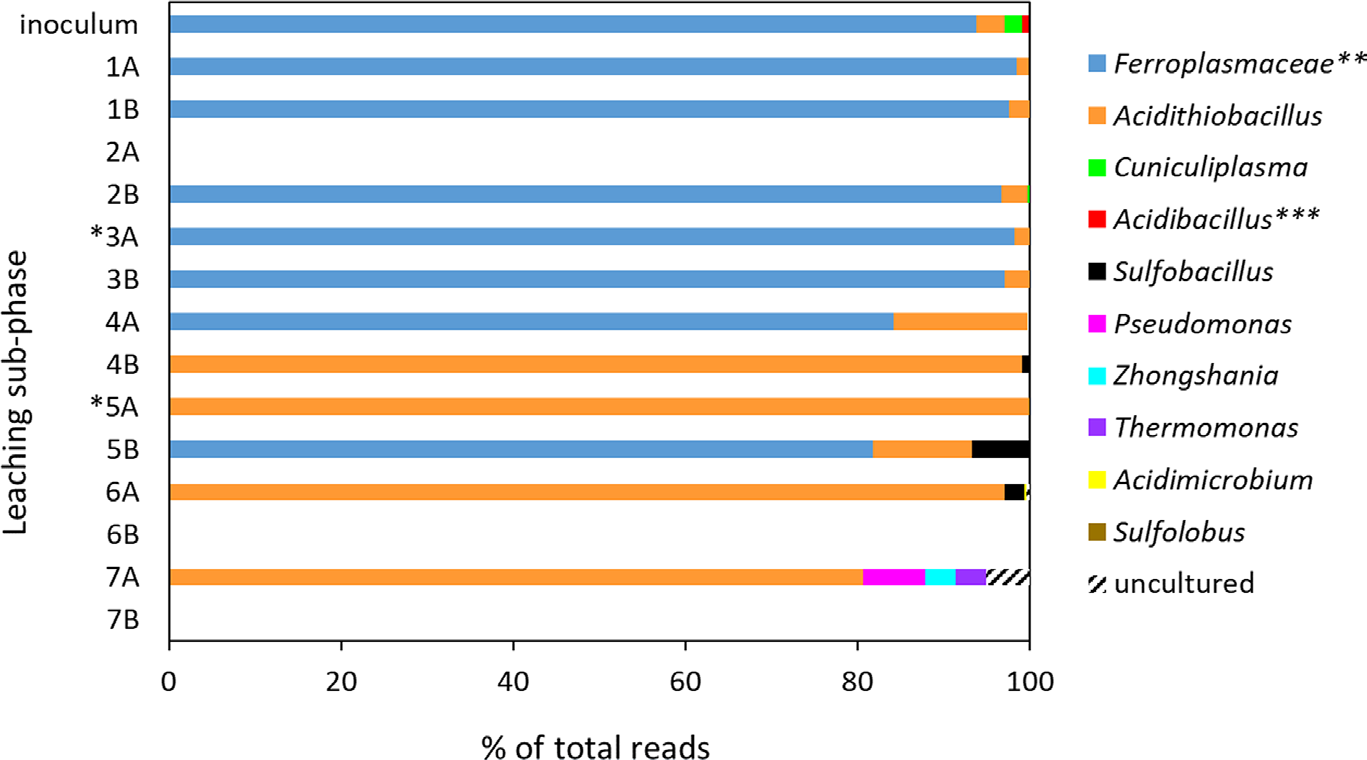
Variations in the composition of the prokaryotic populations in AGB lixiviants (sub-phase A) and column leachates (sub-phase B) during closed-loop LCO leaching, compared to the composition of the inoculum used. A cut-off of 0.1 % of total reads was used for all samples. *, sub-phases that commenced with recirculation of Co-stripped liquors. **, higher taxa that were pooled with respective genera. The genus ‘*Acidibacillus*’ has recently been reclassified as *Sulfoacidibacillus* and *Ferroacidibacillus* [64]. Missing data in the chart are due to unsuccessful DNA amplification.

The diversity of the original consortium (reported in Table 2) was significantly reduced during adaptation, with only a few taxonomic groups detected in the adapted culture used as inoculum for closed-loop indirect leaching. Fig. 4 shows that 93.8 % of total reads were *Ferroplasmaceae*, 3.3 % *Acidithiobacillus,* 2.0 % *Cuniculiplasma*, and 0.8 % ‘*Acidibacillus*’ (recently renamed as *Sulfoacidibacillus* and *Ferroacidibacillus* by Johnson *et al*. [64]). The microbial community greatly varied during the closed-loop leaching (Fig. 4): samples from phases 1–4 and 5B were highly dominated by *Ferroplasmaceae* (81.7–98.5 % of total reads), with low proportion of *Acidithiobacillus* (1.4–3.0 % of total reads), while the rest of the samples were dominated by *Acidithiobacillus* spp. (>80.6 %), with minor proportions of *Sulfobacillus* and a few contaminating genera.

### Generation of Co nanoparticles from bioleachates

Cultures of the SRB *D. alaskensis* were resuspended in MOPS buffer to remove excess extracellular biogenic H_2_S and control precipitation with slow release from sulfate metabolism. This resulted in selective precipitation of Co, while only negligible Li removal (≤1.9 %) was observed in the bioprecipitation assays. All dissolved Co was removed from bioleachates containing up to 110 mg l^−1^ Co, but the recovery rate decreased with increasing Co content in the bioleachates (with only 15.3 % being recovered from a bioleachate containing 1194 mg l^−1^ Co) (Table 7). In abiotic controls, removal of both Co and Li was negligible.

**Table 7.** Cobalt nanoparticle precipitation by *Desulfovibrio alaskensis* G20: initial contents and removal rates of dissolved Co and Li from amended LCO bioleachates (Fe-free, diluted in water) via precipitation with biogenic H_2_S

Amended bioleachate	Metal content (mg l^−1^)*		Co removal (%)		Li removal (%)	
	Co	Li	Biotic	Abiotic	Biotic	Abiotic
A	1194.2	241.0	15.3	1.7	0.0	0.0
B	109.7	24.3	100	0.0	1.9	0.0
C	289.1	96.8	57.6	0.0	0.0	0.0
D	18.0	10.5	100	0.0	0.0	0.3

*, dissolved metal contents in the bioprecipitation assays were 10-fold diluted compared to those in the initial amended bioleachates.

**Fig. 5.**
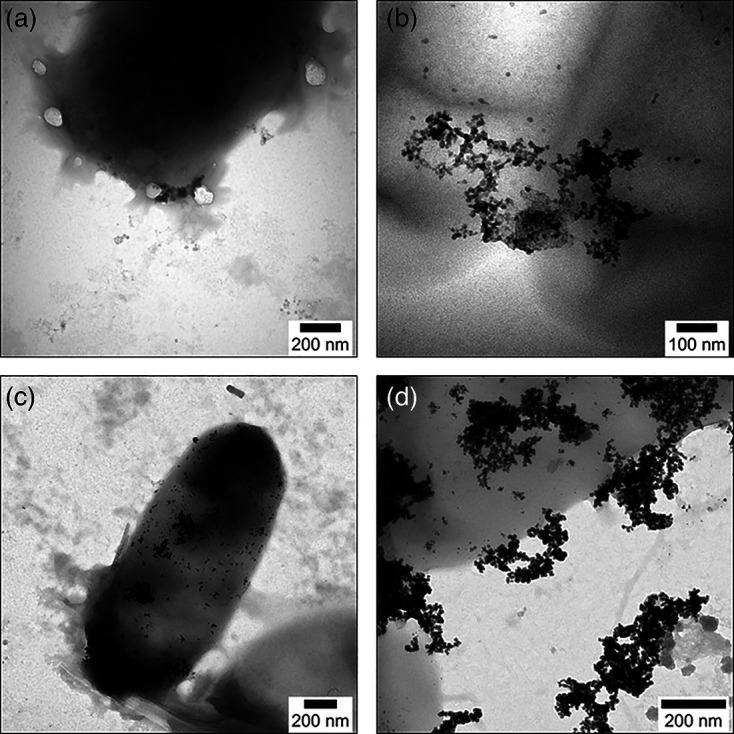
TEM images: (a) 35 000, (b) 70 000, (c) 17 000 and (d) 55 800× magnification of Co nanoparticles generated via reaction of Co dissolved in amended bioleachates with biogenic H_2_S produced by *Desulfovibrio alaskensis* G20. Cobalt nanoparticles are shown as black structures attached to the bacterial cell surfaces. Initial metal composition of bioleachates (a), (b), (c), and (d) is shown in Table 7.

Dense nanoparticles were produced on the surface of *D. alaskensis* cells in the biotic assays (Fig. 5), indicating that bioprecipitated Co is often attached to cell membranes.

**Fig. 6.**
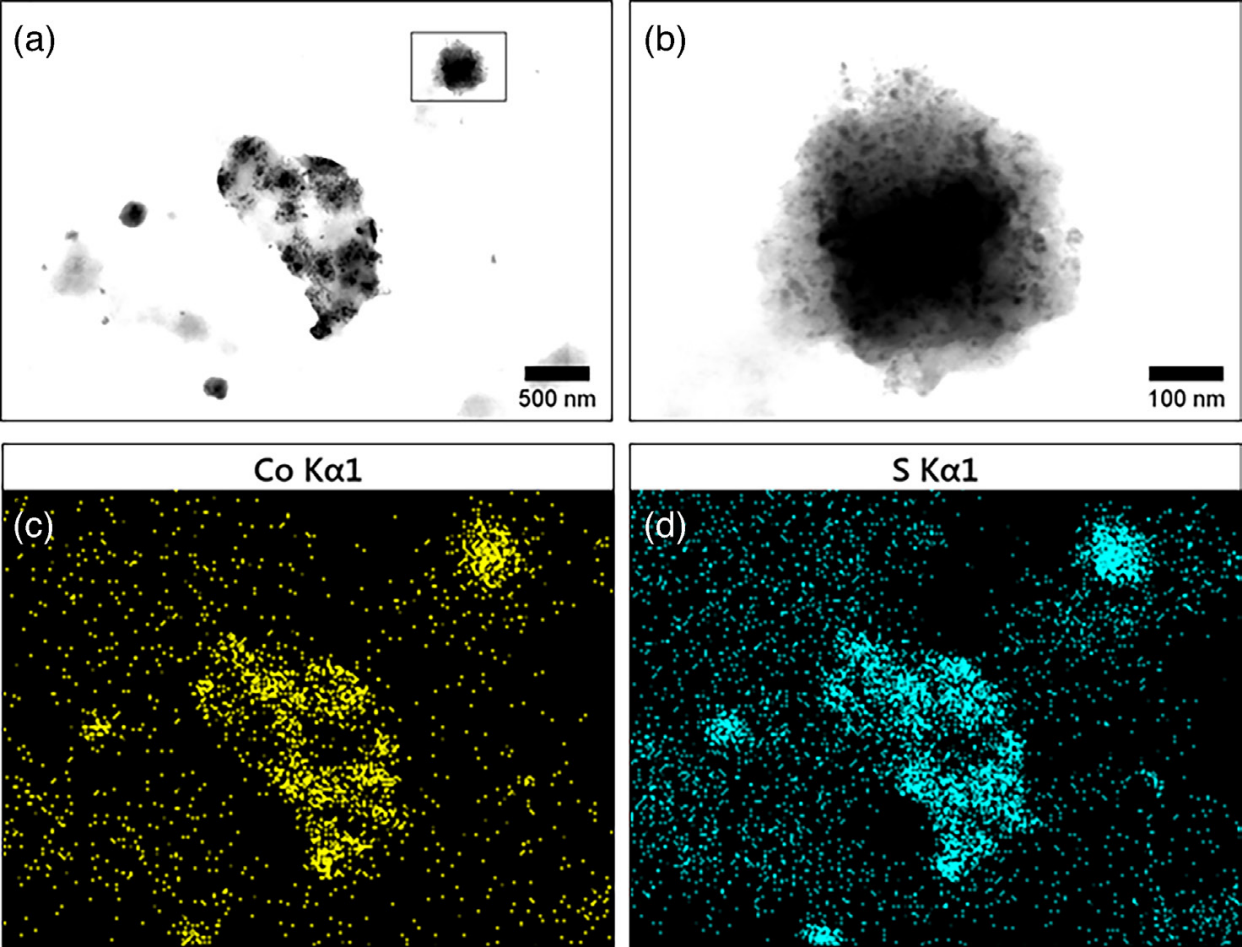
(**a**) STEM image (25 000× magnification) of *D. alaskensis* treated with an amended LCO bioleachate (containing 281.9 mg l^−1^ Co; bioleachate C in Table 7), (**b**) enlarged image of an extracellular nanoparticle, and EDX maps of the same cell for Co (**c**) and S (d).

The results of EDX analysis of a sample collected from the assay containing bioleachate C (281.9 mg l^−1^ Co; Table 7) confirmed that the extracellularly deposited nanoparticles were Co sulfide-based (Fig. 6).

## Discussion

LCO dissolves in an acidic environment according to Equation 1 [65]:

4LiCoO_2_+12H^+^ → 4Li^+^ + 4Co^3+^ + 6H_2_O + O_2_ (1)

Released Co^3+^ is reduced to Co^2+^ by H_2_O, with the reaction rate decreasing with increasing acidity [66], which has practical implications for bioleaching processes – Fe^2+^ can act as a reducing agent in bioleaching systems, increasing Co dissolution from LIBs (Equation 2 [29]):

2FeSO_4_+2LiCoO_2_+4H_2_SO_4_ → Fe_2_(SO_4_)_3_ + 2CoSO_4_+2Li_2_SO_4_+4H_2_O (2)

It could be convenient for LIB bioleaching applications that microbial oxidation of Fe^2+^ is completely inhibited at pH <1.2 [67]. Additionally, *Acidithiobacillus* spp. that oxidize S (but not Fe^2+^) have been shown to facilitate Fe^3+^ reduction at pH <1.0 even in the presence of O_2_ [68], regenerating Fe^2+^ that can subsequently accelerate Co^3+^ reduction to Co^2+^ [34]. Iron can therefore act as a redox catalyser in extremely acidic liquors, even in small amounts, which prevents massive Fe precipitation and passivation of LIB surface. Very low pH values are difficult to achieve in bioleaching systems with LIBs, due to the alkaline nature of the batteries. In this study, additions of 20 mM Fe^2+^ were used in direct bioleaching systems, forming Fe precipitates in the initial phases of the experiments (when pH >2.0), which dissolved in later stages. Iron reduction mediated by S oxidizers at very low pH was expected to occur in the leaching final stages (when pH dropped below 1.0). However, the Fe present was in the form of soluble Fe^3+^, and Fe^2+^ was not detected by the ferrozine assay (and *E*_H_ values ranged from +730 to +810 mV; Fig. S5). During the indirect leaching, lower amounts of Fe^2+^ (1 mM) were supplemented into AGB at the end of each sub-phase A (when pH reached 0.9). No formation of Fe^3+^ precipitates was observed, but the total amount of soluble Fe slowly declined throughout the initial leaching phases when pH in the leaching column >2.0 (with the initial values as high as 3.8, decreasing gradually in later leaching phases). The changes in *E*_H_ followed this trend, ranging from +440 mV (in the initial leaching phases) to values as high as +800 mV (in later phases when Fe^3+^ was in solution) (Fig. S5).

The lack of Fe^3+^ reduction by acidithiobacilli at low pH was likely due to the presence of the Fe^2+^-oxidizing *Ferroplasma*. The archaeon was detected in high proportions (dominating most samples; Fig. 4), likely introduced into the system as a contaminant of the environmental isolates or with non-sterile LCO. We hypothesize that the dominance of this chemomixotroph in the bioleaching systems (also found in high numbers in e.g. BIOX industrial operations [69]) was due to elevated availability of organic carbon from accelerated cell lysis in the extremely metal-rich system. Hetero- and mixotrophic microorganisms can detoxify the environment by consuming organic carbon compounds, to which acidophilic chemolithotrophs are susceptible [70,71]. The presence of the mixotrophic *Ferroplasma* in a battery leaching system, therefore, presented a significant advantage, enabling generation of biogenic H_2_SO_4_ by the S-oxidizing *At. caldus* and *At. thiooxidans*. Interestingly, the moderate thermophile (with a temperature optimum of 45 °C) *At. caldus* thrived at 32 °C. Slight variations in AGB operation temperature (from 35 °C in phase 1 to 30 °C in phase 2, and 32°C in phases 3 to 7) did not seem to correlate with the consortium evolution (Fig. 4) or metal leaching rate. This was ascribed to complex interactions within microbial consortia, which have generally been shown to be more robust and successful in bioleaching operations compared to individual species [21,72, 73]. Mesophilic heterotrophs were detected as minor contaminants in the adapted consortium used for indirect closed-loop leaching: the recently described bacterial genera *Sulfoacidibacillus* and *Ferroacidibacillus* (that oxidize Fe^2+^ and in the case of some species also S; *Firmicutes* [64]), and archaeon *Cuniculiplasma* (*Thermoplasmatales* [74]). The above genera utilize organic carbon, but they quickly disappeared from the consortium and were not detected during the indirect leaching.

The moderately thermophilic direct bioleaching system was highly dominated by the genus *Sulfobacillus* (with a minor proportion of contaminating *Leptospirillum*). *Sb. thermosulfidooxidans* and *Sb. acidophilus* grow autotrophically and mixotrophically on Fe^2+^, on S^0^ in the presence of yeast extract, and heterotrophically on yeast extract. Autotrophic growth on S^0^ was consistently obtained only with *Sb. acidophilus* [75]. The moderately thermophilic consortium showed lesser performance during LCO leaching, possibly due to a higher pH minimum (~1.5) of *Sulfobacillus* spp. High metal recovery rates (~90 % Co and Li) were only obtained using the non-adapted moderately thermophilic consortium during direct bioleaching of very low LCO pulp densities (0.2 %). However, the recovery rates decreased significantly with increasing LCO pulp density. While adaptation of the consortium to elevated LCO pulp densities did not prove efficient, an application of a two-step process (with a delayed addition of LCO) slightly improved the recovery rates (to 45 % Co and 55 % Li at 3 % LCO). The adaptation process proved more successful with the mesophilic consortium, yielding 72.5 % Co and 92.9 % Li at 3 % LCO, and the application of a two-step process for the direct bioleaching further improved the recovery rates (to 82.5 % for Co and 98.7 % for Li).

Diverse metal recovery rates have been reported by other researchers using acidophilic chemolithotrophs or organic acid-producing fungi (summarized in Table 1). The vast majority of published works used a flask set-up and direct bioleaching to extract metals from low pulp densities of battery materials (≤1 %). A few studies have used adaptation to elevated pulp densities and two-step bioleaching. Ghassa *et al*. [29] reported high metal extraction (>99 % Co and Ni and 84 % Li) using an adapted moderately thermophilic consortium containing four acidophiles, but a low pulp density (1 %) of spent battery material was used, and extensive Fe^3+^ precipitation was reported. Two-step bioleaching using a *Leptospirillum ferriphilum*-dominated consortium was successfully applied to leach >92 % Cu, Zn, Ni, and Co, and 38 % Li from 1 % spent LIBs by Khatri *et al*. [31]. Other studies used a two-step process, but with adapted mixed cultures of acidophilic chemolithotrophs: Lalropuia *et al*. [76] leached 100 % Al, Co, Li, Mn, and Ni from 1 % black mass using an environmental enrichment dominated by *Acidithiobacillus* and *Alicyclobacillus* spp., and Heydarian *et al*. [30] reported 50.4, 99.2, and 89.4 % recovery of Co, Li, and Ni, respectively, from 4 % spent LIBs using *At. ferrooxidans* and *At. thiooxidans*. Considering metal recovery rates (particularly that of Co) as well as battery material pulp densities, superior results were obtained during direct bioleaching with the adapted mesophilic consortium in this study.

Despite improvements, direct bioleaching is generally limited to low pulp densities. To overcome this process limitation, Khatri *et al*. [31] used a spent medium; between 19 and 78 % of the metals were extracted at 1 % pulp density, but the extraction efficiencies decreased significantly with increasing battery loads, yielding only 5–39 % metal extraction rates at 10 % pulp density. Do *et al*. [40] reported 86–92 % metal recovery from 10 % spent LIBs in three 2 h cycles using a biogenic Fe^3+^-rich lixiviant generated by *At. ferrooxidans*. However, the authors claim 0.5 M H_2_SO_4_ was produced during pre-cultivation of the bacterium, without reporting any S substrate being present and disregarding the fact the pH minimum of *At. ferrooxidans* is ¬1.3 (while 0.5 M H_2_SO_4_ has pH 0.3). Boxall *et al*. [56] applied indirect bioleaching with biogenic Fe^3+^ and/or H_2_SO_4_ (generated by *At. ferrooxidans* and *At. thiooxidans*) to extract metals from LIB waste. Low yields (< 10 %) were improved by applying several subsequent leaching stages, achieving 53.2 % Co, 60 % Li, 48 % Ni, 82 % Mn, and 74 % Cu recovery rates. Slightly improved Co recovery (~58 %) and significantly improved Li recovery (100 %) were obtained in this study. The metals were continuously leached with biogenic H_2_SO_4_ in several phases, during which the leachates were recirculated to achieve dissolved Co concentrations suitable for offline recovery (but not inhibitory to the leaching consortium). Most works that have addressed metal extraction and subsequent solubilized metal recovery have used harsh chemical leaching [55] and, to our knowledge, only Biswal *et al*. [25] and Do *et al*. [40] used microorganisms. Additionally, no closed-loop system involving biosolubilization and recirculation of metal-stripped liquors has been described before.

Several techniques can be applied to recover metals from solution. Due to its simplicity, low cost and suitability for closed-loop systems, selective precipitation was selected to recover Co from the leachates in this study. Dissolved Co was precipitated from the leachates using NaOH and high recovery rates (>99.8 %) were achieved. The dissolved Co was present as hexaaquacobalt(II) ion [Co(H_2_O)_6_]^2+^ in the pink bioleachates, and was recovered as a green–blue precipitate of [Co(H_2_O)_4_(OH)_2_] on reaction with NaOH (Equation 3):

[Co(H_2_O)_6_]^2+^ + 2OH^−^→ [Co(H_2_O)_4_(OH)_2_] + 2H_2_O (3)

Cobalt hydroxides can be oxidized to Co_3_O_4_ by calcination (e.g. [77]) and used for LCO synthesis [78]. The dissolved Li could be recovered as carbonate [79] or phosphate [80]. The above-mentioned compounds are precursor materials for a thermal synthesis of LCO [81]. Therefore, the presented closed-loop system generated products suitable for battery supply chains, while significantly lowering amounts of liquid waste(s), which were also less hazardous compared to wastes generated by other processes.

In addition, dissolved Co was recovered from the bioleachates as high-purity metallic Co by electrodeposition onto carbon felt and stainless steel. Maintaining the electrolyte pH was necessary to achieve high current efficiencies at the low Co concentration in bioleachates (~4 g l^−1^). For improved pH control, a divided flow cell could be used, which would maintain a stable pH at the cathode, whilst a feed and bleed system would maintain Co concentration in the electrowinning cell loop, as developed by SINTEF [82]. The deposited Co could be recovered by mechanical removal from the stainless steel and by smelting in a furnace from the carbon felt. Another possible option would be using the Co-coated electrode as an anode in an electrochemical cell, in which the deposited Co would redissolve to form a more concentrated electrolyte. This way the carbon felt could be reused, and a very pure Co deposit would be recovered from the enriched high-concentration electrolyte. The main advantage of recovering Co from bioleachates via electrowinning is the lower consumption of NaOH for pH adjustments compared to precipitation. This reduces process costs as well as Na^+^ concentration in raffinates that could otherwise have inhibitory effects on leaching microbes.

As a last method, Co nanoparticles were generated from the bioleachates using the SRB *D. alaskensis* which produced biogenic H_2_S from the sulfates supplied in the bioleachate. Microbially catalysed precipitation of metals from LIB leachates has previously been demonstrated. Calvert *et al*. [83] used an SRB consortium to generate low-value mixed metal precipitates, which could be used for the production of steel or could be further purified for other applications. Removal of Co from LIBs generated via hydrometallurgical processing has also been shown using *D. alaskensis*, but, to our knowledge, recovery of Co nanoparticles from microbially generated leachates has not been described before, and the presented results serve as a proof of concept.

## Conclusions

S^0^-enhanced bioleaching of a LIB cathode material was performed using mesophilic and moderately thermophilic acidophilic consortia. S^0^ is an abundant and cheap waste product, and its bio-oxidation provides H_2_SO_4_ for sustainable recycling of waste LIBs. Solubilization of Co and Li from 0.5 and 3 % LCO was investigated using non-adapted and adapted consortia in one- and two-step flask experiments. The moderate thermophiles performed better at low pulp densities, while mesophiles were more successful at elevated LCO loads. The adapted mesophilic prokaryotic consortium was used for indirect leaching in a closed-loop system connecting a bioreactor and leaching column, achieving high metal recovery rates (58 % Co and 100 % Li) at 10 % LCO, providing high-purity Co hydroxide products, while minimizing waste generation. Besides precipitation, electrowinning was optimized for Co recovery from bioleachates that contain significantly lower concentrations of dissolved Co than liquors produced by conventional hydrometallurgical approaches. In addition, Co nanoparticles were generated from the bioleachates using SRB.

In summary, an improved bioleaching-based technology for Co recovery from LIBs was developed. This sustainable closed-loop system is suitable for scale-up and has the potential to be adapted to other battery chemistries. Moreover, the Co extraction rates could be further improved by mixing the solid residues with a fresh Li-bearing battery material. Different routes for Co recovery from solution provided high-quality products suitable for a battery supply chain and other applications.

## supplementary material

10.1099/mic.0.001475 Uncited Supplementary Material 1.

## Abbreviations

AGB: acid-generating bioreactor
At.: Acidithiobacillus
CA: cellulose acetate
DRC: Democratic Republic of Congo
EDX: energy dispersive X-ray spectroscopy
ICP-OES: inductively coupled plasma atomic emission spectroscopy
L.: Leptospirillum
LCO: lithium cobalt oxide
LIBs: lithium-ion batteries
MOPS: 3-(N-morpholino)propanesulfonic
OD: optical density
Sb.: Sulfobacillus
SEM: scanning electron microscopy
SRB: sulfate-reducing bacteria
STEM: scanning transmission electron microscopy
SX: solvent extraction
TEM: transmission electron microscopy
XRF: X-ray fluorescence
