# Supplementary material for: A novel closed-loop biotechnology for recovery of cobalt from a lithium-ion battery active cathode material
Source: Microbiology (Reading). 2024 Jul 17;170(7):001475. doi: 10.1099/mic.0.001475 (PMC11318048; doi:10.1099/mic.0.001475)
Supplement: Uncited Supplementary Material 1. [file mic-170-01475-s001.pdf]

Supplementary Materials for

A novel closed-loop biotechnology for recovery of cobalt from a lithium-ion battery active cathode material

Eva Pakostova[1,2,3,4]*, John Graves[2], Egle Latvyte[2], Giovanni Maddalena[5,6], Louise Horsfall[5,6*]

[1]Centre for Health and Life Sciences, Institute of Health and Wellbeing, Coventry University, Coventry, CV1 5FB, UK

[2]Centre for Manufacturing and Materials, Institute for Clean Growth and Future Mobility, Coventry University, Coventry, CV1 5FB, UK

[3]MIRARCO Mining Innovation, Sudbury, ON P3E 2C6, Canada

[4]Goodman School of Mines, Laurentian University, Sudbury, ON P3E 2C6, Canada

[5]School of Biological Sciences, University of Edinburgh, Edinburgh, EH9 3FF, UK

[6]Faraday Institution (ReLiB project), Quad One, Harwell Science and Innovation Campus, Didcot, UK

*Corresponding authors: EP: 150560@mail.muni.cz, LH: louise.horsfall@ed.ac.uk

Keywords: Bioleaching; Lithium-ion batteries; Closed-loop metal recycling; Cobalt electrowinning; Nanoparticles.

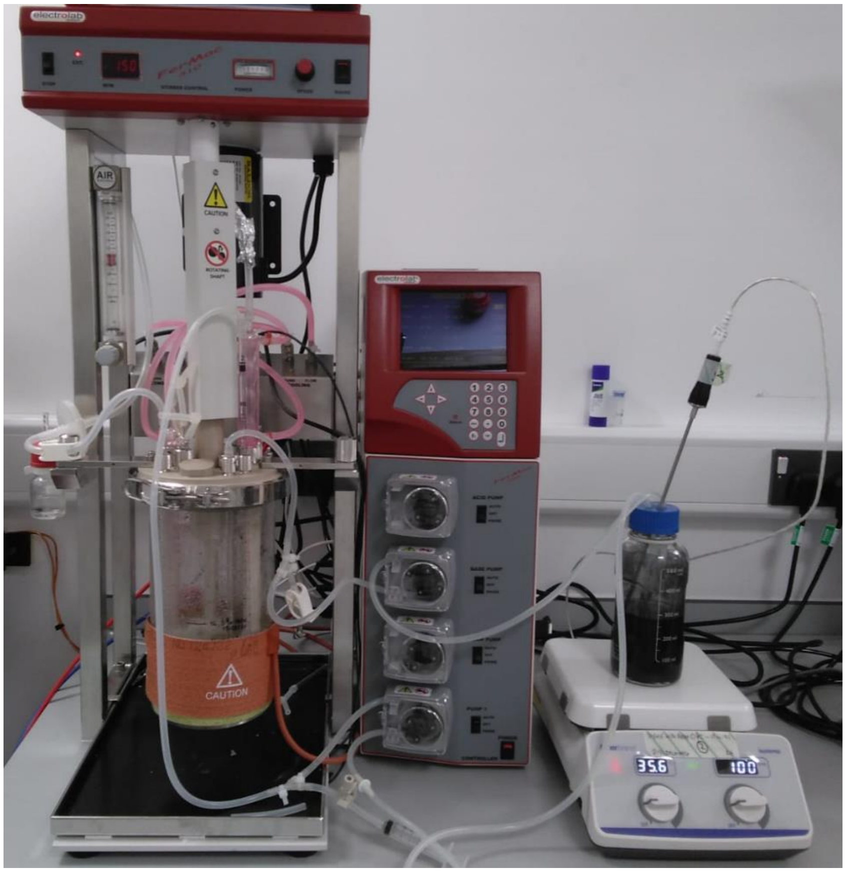

Figure S1. A laboratory set-up for closed-loop indirect leaching of LCO using an acidophilic prokaryotic consortium.

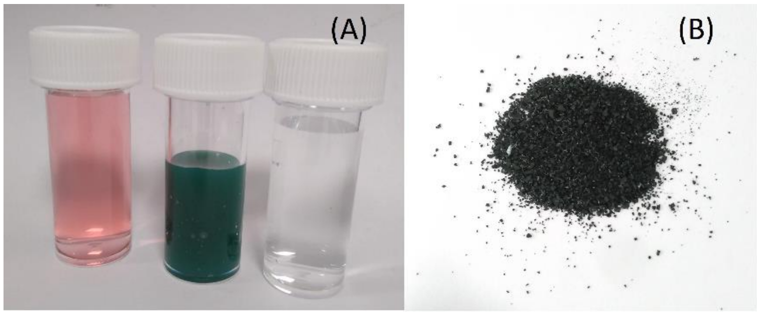

Figure S2. Selective precipitation of Co (using NaOH) from bioleachates generated during closed-loop indirect LCO leaching using acidophilic prokaryotes: (A) comparison of Co-rich leachate (pink), leachate with pH adjusted to ~9.5 containing Co hydroxide(s) in solution (green), and raffinate after Co hydroxide filtration (clear); (B) washed, dried, and crushed Co product.

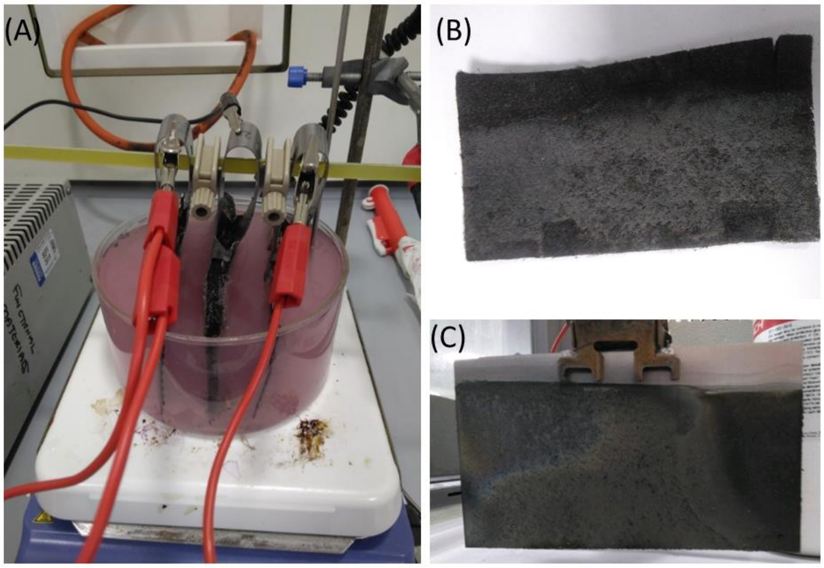

Figure S3. Electrowinning of Co from bioleachates generated during closed-loop indirect LCO leaching using acidophilic prokaryotes: (A) electrowinning cell with a carbon felt cathode between two mixed metal oxide anodes; (B) carbon felt cathode with plated Co (after 2 hours of deposition); and (C) stainless steel cathode with plated Co (after 12 minutes of deposition).

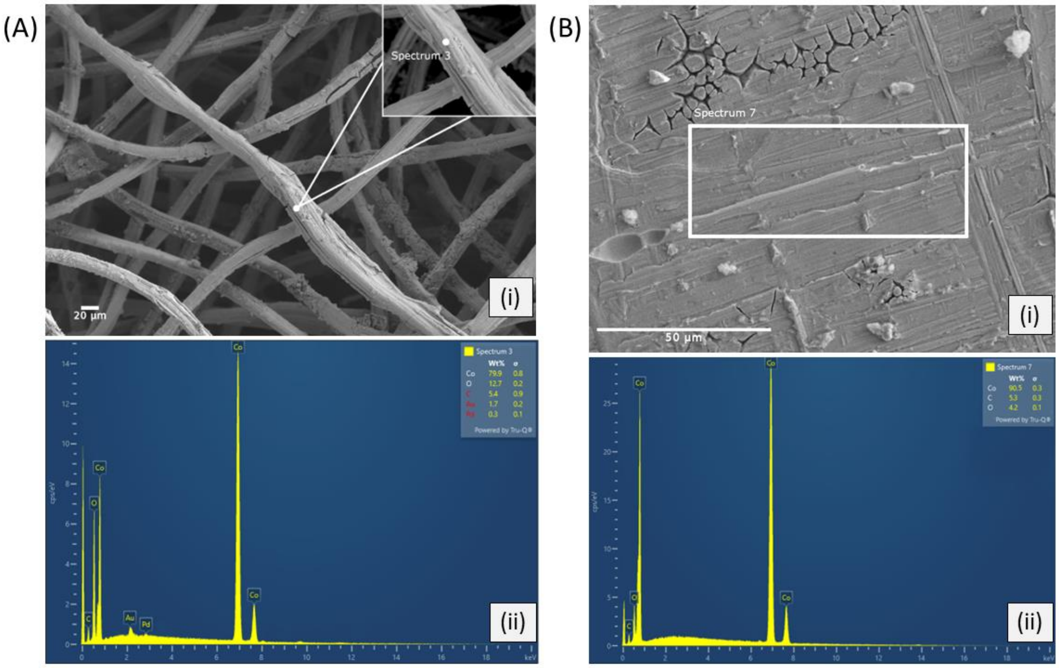

Figure S4. SEM (i) and EDS (ii) results of Co electrodeposited on (A) carbon felt and (B) roughened stainless steel, recovered at 30 mA cm^{-2} and 70°C from bioleachates adjusted to pH 4.

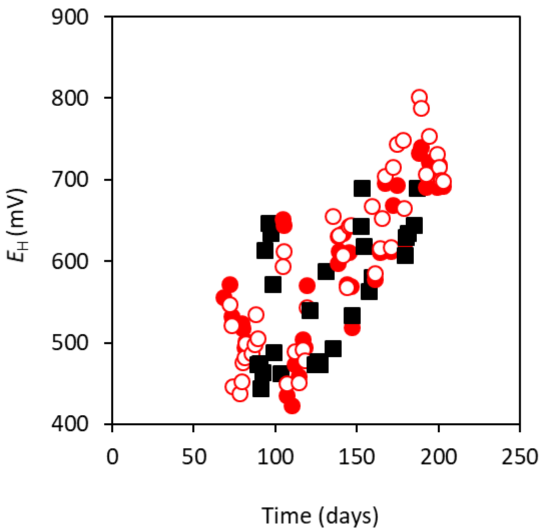

Figure S5. Changes in redox potential (E_H) during closed-loop indirect bioleaching of LCO using prokaryotic consortia previously adapted to LCO: sub-phases A in AGB (■), and sub-phases B in AGB (●) and leaching column (○). For more about the process sub-phases, see Section 2.3.
